# The Evolution of Flexible Electronics: From Nature, Beyond Nature, and To Nature

**DOI:** 10.1002/advs.202001116

**Published:** 2020-08-28

**Authors:** Panpan Wang, Mengmeng Hu, Hua Wang, Zhe Chen, Yuping Feng, Jiaqi Wang, Wei Ling, Yan Huang

**Affiliations:** ^1^ State Key Laboratory of Advanced Welding and Joining Shenzhen 518055 China; ^2^ Flexible Printed Electronic Technology Center Shenzhen 518055 China; ^3^ School of Materials Science and Engineering Shenzhen 518055 China

**Keywords:** energy storage, flexible electronics, flexible sensors, functional designs, nature

## Abstract

The flourishing development of multifunctional flexible electronics cannot leave the beneficial role of nature, which provides continuous inspiration in their material, structural, and functional designs. During the evolution of flexible electronics, some originated from nature, some were even beyond nature, and others were implantable or biodegradable eventually to nature. Therefore, the relationship between flexible electronics and nature is undoubtedly vital since harmony between nature and technology evolution would promote the sustainable development. Herein, materials selection and functionality design for flexible electronics that are mostly inspired from nature are first introduced with certain functionality even beyond nature. Then, frontier advances on flexible electronics including the main individual components (i.e., energy (the power source) and the sensor (the electric load)) are presented from nature, beyond nature, and to nature with the aim of enlightening the harmonious relationship between the modern electronics technology and nature. Finally, critical issues in next‐generation flexible electronics are discussed to provide possible solutions and new insights in prospective exploration directions.

## Graphical abstract

The flourishing development of multifunctional flexible electronics cannot leave the beneficial role of nature. Frontier advances on flexible electronics including the main individual components (i.e., energy (the power source) and the sensor (the electric load)) are presented from nature, beyond nature, and to nature. Critical issues are also discussed to provide possible solutions and new insights in prospective directions.

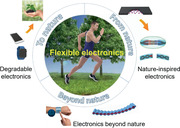

## 1 Introduction

The advent of flexible electronics have brought infinite varieties for their powerful penetration into many fields of smart electronics including artificial e‐skin,^[^ ^1^, ^2^, ^3^, ^4^ ^]^ flexible touch sensors,^[^ ^5^ ^]^ health monitors,^[^ ^6^, ^7^, ^8^, ^9^ ^]^ implantable devices,^[^ ^10^, ^11^ ^]^ and so forth.^[^ ^12^, ^13^, ^14^, ^15^, ^16^ ^]^ Simultaneously, great challenges were generated that primarily derived from the growing demands on more advanced functionality of flexible electronics.^[^ ^17^, ^18^ ^]^ Next‐generation flexible electronics should be developed with excellent mechanical deformability as well as integrated functionality to fulfill the requirement of diversified application markets.^[^ ^19^, ^20^, ^21^, ^22^, ^23^, ^24^ ^]^ New materials and approaches must be explored to break through the limits of conventional methodologies. Fortunately, various living organisms existing in nature possess many multi‐functional materials as well as special environment‐adapted structures during the long evolution history,^[^ ^25^, ^26^, ^27^, ^28^ ^]^ providing us with valuable cues to resolve the challenges to endow flexible electronics with unimaginable functionalities.^[^ ^29^, ^30^, ^31^ ^]^

Specifically, functional materials in flexible electronic devices should possess excellent mechanical properties to accommodate large strain/stress or geometrical deformation in addition to their basic functionality such as energy generation, energy storage, signal sensing, etc.^[^ ^32^ ^]^ Innovative structural designs that stem from the existing natural structure and as‐resulted function could bring new concepts for smart performances.^[^ ^33^, ^34^ ^]^ For instance, high adhesion and water resistance are highly demanded for wearable and skin‐attachable electronics. Inspired by the protuberance or infundibulum in octopus suckers, an artificial patch with octopus‐like pattern was presented with the highly adhesive capabilities under both dry and wet condition on various surfaces,^[^ ^35^ ^]^ which is a promising approach to realize the wearable and skin‐attachable sensors for in vitro and in vivo monitoring of biosignals.

For most electronics, energy storage is an indispensable component as their power sources.^[^ ^36^, ^37^ ^]^ The incorporation of flexibility, stretchability, and compressibility in energy devices would provide human‐friendly interfaces with conformal contact capability.^[^ ^38^, ^39^, ^40^, ^41^ ^]^ The self‐healing ability, inspired by the spontaneous restorative feature of human skin, endow the flexible devices with self‐repairing ability upon damages as well as enhancing their wearability. The environmental sensing ability, one of the most widely used functions in many flexible electronics devices to monitor atmosphere condition, are also derived from commonly existed nature signals, such as light,^[^ ^42^ ^]^ humidity,^[^ ^43^, ^44^, ^45^ ^]^ pH,^[^ ^46^ ^]^ etc. For the flexible electronics, functionality integration with various powerful functionalities including self‐powering, energy storage, sensing, mechanical deformability, etc., was rather essential to cater for more complicated condition in human‐interactive devices, soft robots, medical devices, etc.^[^ ^47^, ^48^, ^49^, ^50^, ^51^, ^52^, ^53^ ^]^ Inspired by triboelectrification, a common physical phenomenon in the nature, triboelectric nanogenerators allow independent operation of many integrated flexible electronic systems such as self‐powered and versatile multimodal sensor systems with functionalities beyond nature.^[^ ^54^, ^55^, ^56^ ^]^

The widespread application of smart electronics has brought radical revolution to human lifestyle but resulted in the rapidly growing electronic waste and environmental issue. To this end, biocompatible and biodegradable electronics have inspired great research interest, which can eventually go to nature by physical vanishment once they have served their purposes.^[^ ^57^ ^]^ In addition, biodegradability allows implanted electronics dissolved or resorbed by the body, greatly reducing the risk of infection by secondary surgery for device removal in clinical applications.^[^ ^58^ ^]^ In view of the above‐mentioned close relationship between the flexible electronics and nature, key research advancements achieved in this emerging research frontier are necessary to be summarized from the aspect of from nature, beyond nature and to nature, aimed at enlightening future directions and establishing economical routes for the design of next generation flexible electronics.

**Figure 1:**
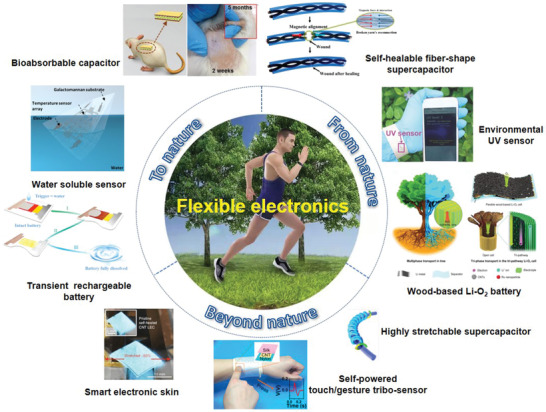
An illustration of the close relationship between flexible electronics and nature from three aspects. From nature: Self‐healable fiber‐shaped supercapacitor. Reproduced with permission.^[^ ^59^ ^]^ Copyright 2015, American Chemical Society. Environmental detecting sensor. Reproduced with permission.^[^ ^60^ ^]^ Copyright 2018, Wiley‐VCH. Tri‐pathway wood‐based Li–O_2_ battery. Reproduced with permission.^[^ ^61^ ^]^ Copyright 2019, Wiley‐VCH. Beyond nature: Highly stretchable supercapacitor. Reproduced with permission.^[^ ^62^ ^]^ Copyright 2018, Springer Nature. Self‐powered touch/gesture tribo‐sensor. Reproduced with permission.^[^ ^63^ ^]^ Copyright 2018, American Chemical Society. Smart electronic skin. Reproduced with permission.^[^ ^23^ ^]^ Copyright 2018, Springer Nature. To nature: Transient rechargeable batteries. Reproduced with permission.^[^ ^64^ ^]^ Copyright 2015, American Chemical Society. Fully water‐soluble sensor. Reproduced with permission.^[^ ^65^ ^]^ Copyright 2018, American Chemical Society. Bioabsorbable capacitor. Reproduced with permission.^[^ ^66^ ^]^ Copyright 2019, Wiley‐VCH.

Herein, we first briefly introduce materials design for flexible electronics, which are mostly inspired from nature with specific functionality realization. Then, we mainly present the frontier advances on flexible energy storage devices (served as power source), flexible versatile sensors (served as electric load) based on the concept that inspire from nature, transcend beyond nature and return to nature with the aim to enlighten the harmonious relationship between green modern electronics technology and our magic nature. A graphical summary of these representative examples to emphasize the design guideline for flexible electronics from nature, beyond nature and to nature are illustrated in **Figure** **1**. Finally, challenges and potential solutions for flexible electronics during realistic applications as well as the new insights in prospective exploration directions are discussed.

## 2 Materials of Flexible Electronics

Exciting achievements have been made in the electronics industry in the last two decades, which is mainly based on conductive, semiconducting, and dielectric materials with micro‐/nano‐engineering procedures.^[^ ^67^, ^68^, ^69^, ^70^, ^71^, ^72^, ^73^ ^]^ For instance, carbon nanotubes (CNTs) are widely employed in microelectronics^[^ ^74^, ^75^, ^76^ ^]^ and energy storage^[^ ^77^, ^78^ ^]^ fields for their superior mechanical strength and excellent electric properties.^[^ ^79^, ^80^, ^81^ ^]^ Zinc oxide (ZnO) with a wide band gap (3.37 eV) as well as distinct electrical, catalytic, and optical properties plays a vital role in sensing,^[^ ^82^ ^]^ energy storage,^[^ ^83^ ^]^ dielectric properties,^[^ ^84^, ^85^ ^]^ photodetecting,^[^ ^86^ ^]^ etc. However, innovative structural designs for these functional materials should be adopted for the construction of flexible electronic devices in order to sustain in various mechanical strain/stress environments and meanwhile remain or even improve their performances.^[^ ^87^, ^88^, ^89^, ^90^ ^]^ A tree‐root‐inspired interlocking system was implemented on ZnO nanowire arrays electrode to enhance both static and dynamic tactile detection sensitivities simultaneously.^[^ ^91^ ^]^ This section would emphasize the recent progress on nature‐inspired materials that are applied in flexible electronics with unique structures and novel functionalities.^[^ ^92^, ^93^, ^94^, ^95^ ^]^

**Figure 2:**
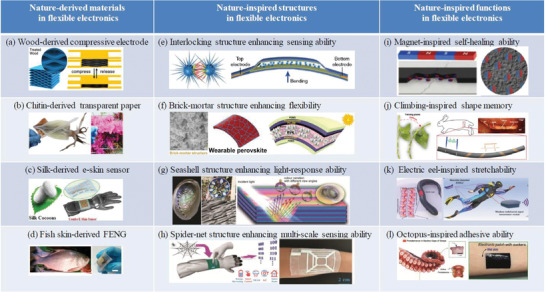
Representative examples of nature‐derived materials, nature‐inspired structures and nature‐inspired functions in flexible electronics. Nature‐derived materials in flexible electronics: a) Wood‐derived compressive electrode. Reproduced with permission.^[^ ^110^ ^]^ Copyright 2018, Elsevier. b) Chitin‐derived transparent paper. Reproduced with permission.^[^ ^92^ ^]^ Copyright 2016, Wiley‐VCH. c) Silk‐derived e‐skin sensor. Reproduced with permission.^[^ ^111^ ^]^ Copyright 2017, American Chemical Society. d) Fish skin‐derived FENG. Reproduced with permission.^[^ ^112^ ^]^ Copyright 2017, American Chemical Society. Nature‐inspired structures in flexible electronics: e) Interlocking structure enhancing sensing ability. Reproduced under the terms of the CC‐BY Creative Commons Attribution 4.0 International License (https://creativecommons.org/licenses/by/4.0).^[^ ^113^ ^]^ Copyright 2018, The Authors, published by Springer Nature. f) Brick‐mortar structure enhancing flexibility. Reproduced with permission.^[^ ^114^ ^]^ Copyright 2019, The Royal Society of Chemistry. g) Seashell structure enhancing light‐response. Reproduced with permission.^[^ ^115^ ^]^ Copyright 2019, Wiley‐VCH. h) Spider‐net structure enhancing multi‐scale sensing. Reproduced with permission.^[^ ^116^ ^]^ Copyright 2019, Wiley‐VCH. Nature‐inspired functions in flexible electronics: i) Magnet‐inspired self‐healing ability. Reproduced with permission.^[^ ^117^ ^]^ Copyright 2016, The Authors, published by American Association for the Advancement of Science. Reprinted/adapted from ref. [117]. © The Authors, some rights reserved; exclusive licensee American Association for the Advancement of Science. Distributed under a Creative Commons Attribution NonCommercial License 4.0 (CC B‐BNC) http://creativecommons.org/licenses/by-nc/4.0/. j) Climbing‐inspired shape memory ability. Reproduced with permission.^[^ ^118^ ^]^ Copyright 2019, The Authors, published by American Association for the Advancement of Science. Reprinted/adapted from ref. [118]. © The Authors, some rights reserved; exclusive licensee American Association for the Advancement of Science. Distributed under a Creative Commons Attribution NonCommercial License 4.0 (CC BY‐NC) http://creativecommons.org/licenses/by-nc/4.0/. k) Electric eel‐inspired stretchability. Reproduced under the terms of the CC‐BY Creative Commons Attribution 4.0 International License (https://creativecommons.org/licenses/by/4.0).^[^ ^119^ ^]^ Copyright 2019, The Authors, published by Springer Nature. l) Octopus‐inspired adhesive ability. Reproduced with permission.^[^ ^35^ ^]^ Copyright 2018, Wiley‐VCH.

In general, the nature‐inspired materials in flexible devices can be mainly divided into two aspects: a) Nature‐derived materials with impressive mechanical flexibility and functionality: These are from renewable and cost‐effective materials in nature, benefitting sustainable development of both electronics and environment.^[^ ^96^, ^97^, ^98^ ^]^ b) Natural structure or function inspired artificial materials or electronics: unique structures and functions in nature inspire the design of new materials and configurations with intriguing property and super performance.^[^ ^99^, ^100^, ^101^, ^102^, ^103^, ^104^, ^105^, ^106^ ^]^ The representative examples of nature‐derived materials, nature‐inspired structures, and nature‐inspired functions in flexible electronics are summarized in **Figure** **2**. For instance, transparent paper made of self‐assembled nanofibers of chitin was demonstrated as a potential substrate for flexible green electronics. A fishskin‐based nanogenerator (FSKNG)/pressure sensor, composed of collagen nanofibrils piezoelectric biomaterials, was demonstrated as wearable and biocompatible sensors for noninvasive and continuous monitoring of human physiological signals. Those flexible electronics derived from natural materials, such as wood, silk, creature, etc., offer a simple yet economical approach to construct sustainable technology owing to their distinguished features of abundance in nature, environmental and human benignity, biocompatibility as well as unique mechanical and electrical properties.

**Figure 3:**
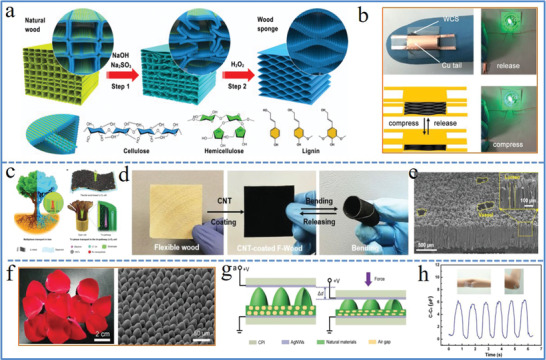
a) Graphical illustration of the structure evolution of natural balsa wood upon chemical treatment. b) Photograph and graphical illustration of the strain sensor connected with a LED light under compression and release conditions. a,b) Reproduced with permission.^[^ ^110^ ^]^ Copyright 2018, Elsevier. c) Schematic illustration of the tree‐inspired tri‐pathway design for flexible Li–O_2_ cells. d) Morphology characterization of the flexible CNT‐coated wood membrane. e) Cross‐sectional SEM images of the CNT‐coated wood membrane showing the multichannel structure. c–e) Reproduced with permission.^[^ ^61^ ^]^ Copyright 2019, Wiley‐VCH. f) Photograph (left) and SEM image (right) of red rose petals by supercritical drying. g) The sensing mechanism illustration of the natural‐material‐based e‐skin. h) The rose‐petal‐based e‐skin with real‐time monitoring of capacitance variations caused by repeated elbow bending. f–h) Reproduced with permission.^[^ ^109^ ^]^ Copyright 2016, The Royal Society of Chemistry.

Nature has provided us with various natural materials that could be used directly after facile treatment, possessing advantages of satisfied performance, easy processability, and eco‐friendliness with wide applications in energy and sensor.^[^ ^107^, ^108^ ^]^ The natural plant with rich channels and hollow structure was the most common examples that could be utilized directly to endow the electronics with various functionalities including compressibility, facilitated transportation ability, sensitivity, etc. For example, a direct wood‐to‐carbon‐sponge transformation was realized via a facile chemical treatment (**Figure** **3**a). The magic transformation from brittle wood carbon with lattice‐like wood structure to compressible wood carbon sponge with spring‐like lamellar structure was achieved by removing lignin and hemicellulose from balsa wood cell walls. The obtained wood carbon sponge exhibits excellent mechanical and electrical sensitive responses, possessing good potential as highly sensitive strain sensors (Figure 3b). Originating from the abundant network of channels for multiphase transport of water, ions, and nutrients in tree trunk, a flexible Li–O_2_ battery was developed that utilized natural wood directly to construct a continuous tri‐pathway structure for ion, O_2_ gas and electron transport (Figure 3c). A rigid and electrically insulating wood membrane was converted to a flexible and electrically conductive electrode (Figure 3d) through facile chemical delignification and CNT coating processes. The multichannel structure of post‐treated wood electrode featured large vessels and small lumina (Figure 3e), greatly facilitating the electrolyte and gas transport simultaneously. The red rose petals with uniform hollow mastoid‐like microstructure arrays (Figure 3f)^[^ ^109^ ^]^ could also be directly used as the dielectric material in flexible capacitive e‐skin after supercritical drying. The natural‐material‐based e‐skin simply consisted of a dried flower petal or leaf sandwiched by two flexible electrodes. Upon loading, the hollow structure was compressed as illustrated in Figure 3g, and thus applied to sense pressures in various cases including the real‐time monitoring the bends generated by elbow movement (Figure 3h), demonstrating its potential in dynamic human motion inspection.

**Figure 4:**
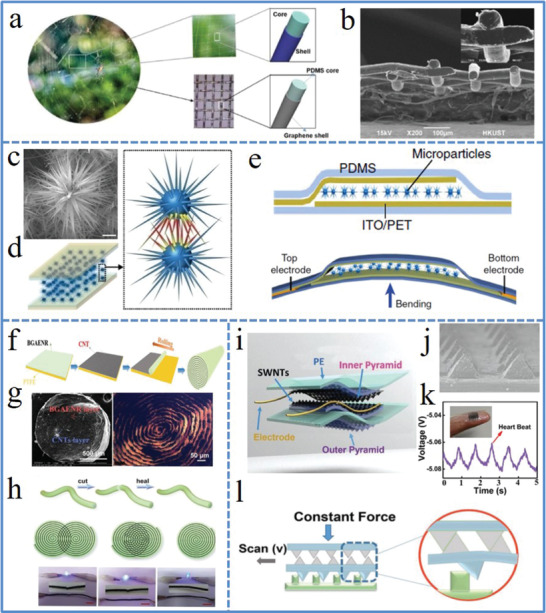
a) Illustration of a spider‐web‐inspired elastomer‐filled graphene woven fabric. b) Cross section of the freestanding graphene woven fabric. a,b) Reproduced with permission.^[^ ^120^ ^]^ Copyright 2019, American Chemical Society. c) SEM image of ZnO particle with sea urchin‐shaped morphology. d) Schematics of a sensor made from a ZnO thin film sandwiched between two electrodes with highlights of the resistance modulation at local spine‐spine sites induced by mechanical stimuli. e) Schematics of the layered configuration in the sensor (top) and the bending state (bottom). c–e) Reproduced under the terms of the CC‐BY Creative Commons Attribution 4.0 International License (https://creativecommons.org/licenses/by/4.0).^[^ ^113^ ^]^ Copyright 2018, The Authors, published by Springer Nature. f) Preparation of spirally layered elastomer. g) SEM (left) and optical images (right) of the elastomer. h) Self‐healing process of the spirally layered elastomer (top) and the reconstruction of conductive path (middle) in a circuit lightening a LED (bottom). f–h) Reproduced with permission.^[^ ^121^ ^]^ Copyright 2019, Wiley‐VCH. i) Schematic configuration of the flexible tactile sensor. j) SEM image of a SWNTs/PDMS film with pyramid microstructure. k) The real‐time heart rate detection by simply attaching the sensor on a finger. l) Schematics of measuring the shear force when a constant force was applied on the sensor with certain scanning velocity. i–l) Reproduced with permission.^[^ ^122^ ^]^ Copyright 2018, Wiley‐VCH.

On the other hand, the unique environment‐adapted structures and functions in nature bring new ideas in the materials design for flexible devices to acquire more excellent performances and promising functionalities. For example, inspired by the highly flexible spider web architecture, an ultrasensitive and wearable strain sensor was proposed based on an elastomer‐filled graphene woven fabric for monitoring human physiological signals (**Figure** **4**a,b).^[^ ^120^ ^]^ Moreover, for biological sensory organelles, where high sensitivity is fairly important, a mechanical sensor based on zinc oxide microparticles with spherically distributed, high‐aspect‐ratio nanospines delivered ultrahigh pressure and strain sensitivity which mimic features of tactile hairs or bristles.^[^ ^113^ ^]^ The tapering spine in ubiquitous mechanosensory organelles of insects not only serves as a lever arm to promote signal transduction, but also protect from mechanical breaking (Figure 4c–e). For the fabrication of self‐healable conductive materials, one typical strategy is to incorporate conductive fillers such as 1D metal nanowires and CNTs into self‐healing matrices, whereas high content of conductive fillers is usually needed for good conductivity, which would suppress the mobility of polymer chains and result in deterioration of the self‐healing capability. Being similar to the Archimedean spiral structure in many plants and animals like sunflower, snail shell, etc., an ultra‐efficiently self‐healable sensor was developed, which could rapidly self‐heal both mechanical (within 15 s) and electrical (within 0.25 s) damages without sacrificing the softness and stretchability of the self‐healing elastomer matrix.^[^ ^121^ ^]^ The spirally structured layout is illustrated in Figure 4f, where alternating CNTs layers (≈2 µm) and elastomer layers (≈20 µm) could be observed clearly (Figure 4g). Interestingly, even if only a small part of the cut sample was reconnected, the reconstruction of conductive pathways is still efficient in such spirally structured layout (Figure 4h). By mimicking the microstructure of our fingerprint, a novel flexible sensor device with improved haptic perception and surface texture recognition was proposed that consisted of single‐walled carbon nanotubes (SWCNT), polyethylene, and polydimethylsiloxane (PDMS) with interlocked and outer micropyramid arrays (Figure 4i,j).^[^ ^122^ ^]^ Due to this unique structure, the sensor exhibited great pressure sensing performance that could real‐time detect heart rate by simply attaching on an index finger (Figure 4k) and also discern the surface texture by gently scanning on different objects (Figure 4l).

## 3 Flexible Energy Harvesting and Storage

All electronics must have power to work. The energy storage, indispensable power supply for most flexible electronics, have attracted significant attention with various smart functionalities that mainly originated from nature. For instance, self‐healable energy storage, fascinated by self‐healing phenomenon of human skin, would prolong the service life for e‐clothes in case of damage.^[^ ^123^, ^124^ ^]^ The electrochromism capability in nature materials has also triggered the advent of electrochromic energy storage devices with appealing applications, such as smart glasses or windows in buildings.^[^ ^125^, ^126^ ^]^ Shape memory materials in nature offer the possibility for smart energy storage devices to memorize and recover their permanent shape in response to external stimuli (such as heat and pressure) and store energy simultaneously.^[^ ^127^, ^128^ ^]^ It is worth mentioning that, by adopting optimal materials or elaborate structural designs, some smart functionalities in flexible energy storage are even beyond nature,^[^ ^129^, ^130^ ^]^ represented by the mechanically stretchable and electrochemically active energy devices with ultrahigh stretchability. On the other hand, energy harvesters could convert ubiquitous mechanical/biomechanical, solar, acoustic, thermal, magnetic energy in our surrounding environment into electricity to power electronic devices continually, which have also been investigated widely as the sustainable energy sources in flexible electronics, such as solar cells, triboelectric nanogenerators (TENGs), piezoelectric generators (PENGs), etc.^[^ ^131^, ^132^, ^133^ ^]^ In this section, key examples of research progress in flexible energy harvesting and storage are selected and summarized from three aspects: self‐healing ability from nature, stretchability/compressibility that beyond nature and biodegradeability to nature, with the purpose to guide their future directions.

### 3.1 Self‐Healing Energy Harvesting and Storage: From Nature

When flexible devices are subjected to practical applications, stress, mechanical damage, accidental cutting, fracture etc., are inevitable during deformations over time. These would seriously lead to limited reliability and lifetime due to the whole breakdown and the excessive generation of electronic wastes as a result.^[^ ^134^ ^]^ Thus, the self‐healing capability, originating from our magic nature such as human skin and Cnidarian hydra which can spontaneously repair damage, is expected to offer impressive advantages for flexible electronics.^[^ ^135^, ^136^, ^137^, ^138^, ^139^, ^140^, ^141^, ^142^ ^]^

**Figure 5:**
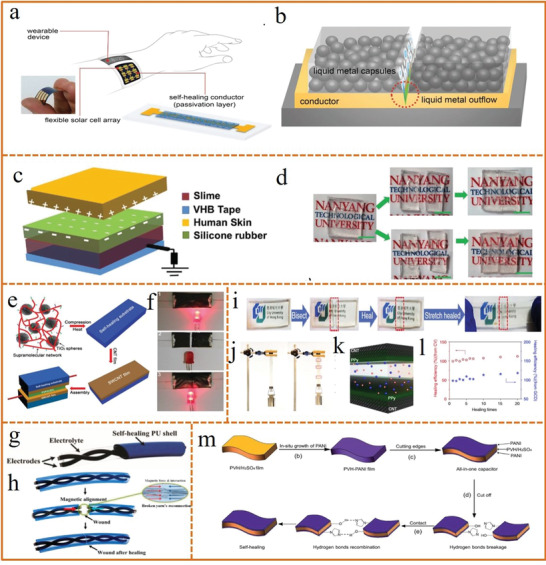
a) Schematic of a solar‐powered smart watch embedded with self‐healing conductor. b) Schematic illustrating the healing mechanism of a metal conductor by a passivation film. a,b) Reproduced with permission.^[^ ^143^ ^]^ Copyright 2018, Wiley‐VCH. c) Schematic diagram of the IS‐TENG. d) Digital photo of the as‐prepared, bifurcated, quadfurcated, and self‐healed IS‐TENG. c,d) Reproduced with permission.^[^ ^144^ ^]^ Copyright 2017, Wiley‐VCH. e) Illustrative fabrication of a self‐healing supercapacitor. f) Photos of self‐healing electrode in a circuit with an LED bulb. e,f) Reproduced with permission.^[^ ^149^ ^]^ Copyright 2014, Wiley‐VCH. g) Schematic components of the self‐healable yarn supercapacitor. h) Schematic illustration of the self‐healing process of the supercapacitor. g,h) Reproduced with permission.^[^ ^59^ ^]^ Copyright 2015, American Chemical Society. i) Demonstration of the self‐healing process of the hydrogel electrolyte. j) Demonstration of the pristine and the self‐healed electrolyte supporting ≈500 g mass, respectively. k) Schematics of the supercapacitor comprising the self‐healing polyelectrolyte and PPy@CNT paper electrodes. l) Healing efficiency under different healing times. i–l) Reproduced under the terms of the CC‐BY Creative Commons Attribution 4.0 International License (https://creativecommons.org/licenses/by/4.0).^[^ ^160^ ^]^ Copyright 2015, Springer Nature. m) Illustrative fabrication of the all‐in‐one self‐healing supercapacitor. Reproduced with permission.^[^ ^161^ ^]^ Copyright 2018, The Royal Society of Chemistry.

Huge efforts have been devoted to fabricating self‐healing energy devices including self‐healing nanogenerators, solar cells, supercapacitors, and batteries, which mainly focus on the function recovery such as conductivity, optical property, electrochemical property, and biological properties as well as the recovery of structural integrity and mechanical property. Typically, the self‐healing mechanisms can be classified into extrinsic and intrinsic self‐healing. For the extrinsic self‐healing, healing agents are necessary which would release and heal the cracks once the damage occurred. Chung et al.^[^ ^143^ ^]^ proposed a self‐healing flexible perovskite solar cells with liquid metal embedded inside a microcapsule as the healing agent, leading to effective recovery of electrical pathways once the microcapsule is broken (**Figure** **5**a,b). For intrinsic self‐healing, hydrogel polymers are commonly used as the self‐healing conductor based on reversible debonding/re‐bonding of different dynamic bonds among polymer chains. Lee et al.^[^ ^144^ ^]^ demonstrated a self‐healing ionic‐skin TENGs using an ionic polymer conductor as the current collector, with the energy‐harvesting performance 12 times higher than that of the silver‐based electronic current collectors, which meanwhile can self‐heal autonomously, quickly, and repeatedly at room temperature (Figure 5c,d).

Most reported self‐healing energy storage devices are based on a self‐healable polymer substrate or shell as an additional component.^[^ ^145^, ^146^, ^147^, ^148^ ^]^ For example, a self‐healing supercapacitor was developed by Chen's group for the first time by coating SWCNT thin film electrodes on a self‐healing supramolecular network substrate (Figure 5e).^[^ ^149^ ^]^ When the electrode suffered physical damage, its conductivity and configuration integrity could be restored by bringing the separated cross sections into reconnection (Figure 5f). Another configuration, a self‐healable yarn‐based supercapacitor was proposed by wrapping the magnetic electrodes with a self‐healable polyurethane (PU) shell (Figure 5g).^[^ ^59^ ^]^ Once the supercapacitor was damaged and an external force moved the broken areas into contact, the PU shell recovered physical integrity and mechanical strength autonomically meanwhile the electrodes recovered their electrical conductivity with the assistance of their own magnetic attraction (Figure 5h). It has to be mentioned that the advent of novel self‐healing electrolyte provides an effective and efficient solution to realize intrinsically self‐healable energy storage,^[^ ^150^, ^151^, ^152^, ^153^ ^]^ that is, self‐healing without the use of any additional component in the device. Such self‐healability is achieved always through reversible interactions including hydrogen bonding,^[^ ^154^, ^155^ ^]^ dynamic borate ester bonding,^[^ ^156^, ^157^ ^]^ coordination,^[^ ^158^ ^]^ covalents,^[^ ^159^ ^]^ etc. Huang and her coworkers reported a self‐healable polyelectrolyte based on a dual crosslinked hydrogel, which could be easily self‐repaired in the ambient condition and the repaired sample displayed mechanical properties as excellent as the pristine one after cycles of breaking/healing (Figure 5i,j).^[^ ^160^ ^]^ A self‐healable supercapacitor was assembled by sandwiching the polyelectrolyte between two PPy@CNT paper electrodes (Figure 5k), which retained the capacitance completely even after 20 breaking/healing cycles (Figure 5l). Additionally, an all‐in‐one device configuration was also regarded to be feasible for the intrinsic self‐healability, which was typically fabricated by in‐situ growth of electroactive components on a free‐standing self‐healable electrolyte as shown in Figure 5m.^[^ ^161^ ^]^

**Figure 6:**
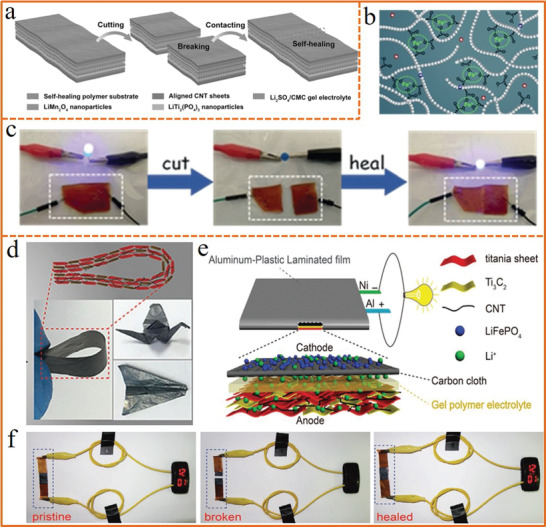
a) Schematic of the self‐healing process of the Li‐ion battery based on self‐healing substrates. Reproduced with permission.^[^ ^166^ ^]^ Copyright 2016, Wiley‐VCH. b) The schematic illustration of the self‐healability origin of PANa‐Fe^3+^ electrolyte. c) Photographs of the PANa‐Fe^3+^ hydrogel serving as anionic conductor successfully connecting the circuit before being cut and after autonomic healing. b,c) Reproduced with permission.^[^ ^167^ ^]^ Copyright 2018, Wiley‐VCH. d) Schematic illustration of the flexible Li ion battery device. e) Schematic of excellent flexibility of the obtained electrode film. f) Photographs of an e‐watch powered by the battery before cutting, after cutting, and after self‐healing. d,e) Reproduced with permission.^[^ ^168^ ^]^ Copyright 2018, Wiley‐VCH.

Compared with self‐healable flexible supercapacitors, the investigation of self‐healable flexible batteries, one of the most important energy storage devices, is still challenging due to more critical requirements on the conductivity of both electrolyte and electrode.^[^ ^162^, ^163^, ^164^, ^165^ ^]^ A flexible and self‐healable aqueous lithium‐ion battery (LIB) was created by Peng et al. for the first time.^[^ ^166^ ^]^ The electrical and mechanical properties of the LIB could be restored after breaking by designing aligned carbon nanotube sheets loaded with electrode nanoparticles on a self‐healing polymer substrate (**Figure** **6**a). This could be defined as extrinsic self‐healing since the self‐healing property was originated from an external self‐healing substrate material. Relatively, the intrinsic self‐healing capability originating from electrode or electrolyte itself could endow the battery with more excellent self‐healing ability. Recently, our group^[^ ^167^ ^]^ designed an intrinsically self‐healable aqueous NiCo//Zn battery by employing a self‐healable sodium polyacrylate (PANa) hydrogel electrolyte based on ferricion‐aided self‐healing mechanism (Figure 6b). The synthesized PANa‐Fe^3+^ hydrogel electrolyte is self‐healable without any stimuli, which could act as an effective ionic conductor to lighten a blue light emitting diode when the broken electrolyte contacted together (Figure 6c), resulting in high level healing efficiency of Zn ion battery (over 87%) after fourth cutting/healing cycle. Furthermore, Wu et al.^[^ ^168^ ^]^ designed a self‐standing electrode with ideal mechanical durability by molecularly coupled 2D titanium oxide and carbide sheets (Figure 6d). Combined with the intrinsic self‐healable poly(ethylene glycol) diamine‐based electrolyte, a self‐healable LIB was achieved (Figure 6e), which could power an e‐watch by simply aligning the two disconnected fragments without external stimulus (Figure 6f). By exploring self‐healing electrode as well as properly designing the electrode structure, the performance of energy devices could be well‐recovered after cutting or damaged. These explorations open up new possibilities for the construction of highly self‐healable energy devices.

### 3.2 Stretchable, Compressible Energy Harvesting, and Storage: Beyond Nature

Stretchability and compressibility are the characteristic features for skin, which are also one necessary function for the practical application of flexible electronics.^[^ ^169^, ^170^, ^171^ ^]^ By employing various novel materials and structural designs, excellent stretchability and compressibility for energy harvesting and storage devices have been achieved with the mechanical performance far beyond nature.^[^ ^172^, ^173^, ^174^, ^175^, ^176^, ^177^ ^]^

**Figure 7:**
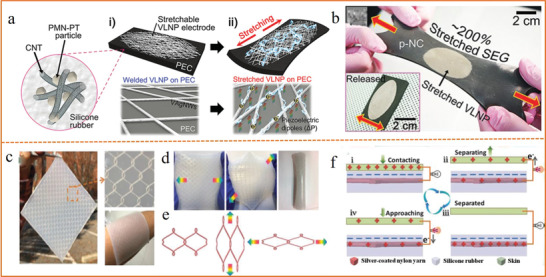
a) Schematic illustration of the hyper‐stretchable elastic‐composite generator. b) The elastic‐composite generator stretched by human hands. a,b) Reproduced with permission.^[^ ^178^ ^]^ Copyright 2015, Wiley‐VCH. c) Photograph image of an actual SI‐TENG and its partially enlarged view. d) Demonstration of the SI‐TENG which can be stretched in any in‐plane direction and be rolled up. e) Schematic demonstrating the in‐plane tensile behaviors of the repeated rhombic unit in the SI‐TENG system. f) Schematic of the operation mechanism of the SI‐TENG. c–f) Reproduced with permission.^[^ ^179^ ^]^ Copyright 2018, Wiley‐VCH.

To achieve the stretchable energy harvesters, all the components should be stretchable. Take TENGs, for example, the stretchability mainly depends on the intrinsic elasticity of the constituent triboelectric layer and the conductor. The metallic based stretchable conductors, such as silver nanowires (AgNWs), gold nanosheets (Au NS), and liquid metal, are the most widely used for TENG due to their well‐connected conducting network, robust mechanical properties and easy fabrication process. Lee et al.^[^ ^178^ ^]^ demonstrate a hyper‐stretchable piezo‐nanogenerator realized by very long Ag nanowires (VAgNWs) stretchable electrodes. This stretchable energy harvester exhibits high stretchability of ≈200% (**Figure** **7**a,b). Besides, non‐metallic ionic conductors based on polymer hydrogel have also emerged as promising deformable electrodes for electronic devices due to their superior stretchability, transparency, healability, and biocompatibility. For those triboelectric layers without good stretchability and triboelectric properties, an alternative route is to use structural patterning (such as origami, kirigami patterns, ladder‐shaped, fabrics, or textiles networks) to achieve stretchability. Wang et al.^[^ ^179^ ^]^ developed a stretchable and conformable TENG constructed from zigzag arranged three‐ply‐twisted silver‐coated nylon yarn embedded into flexible silicone rubber elastomer, which exhibited desired stretchability (30%), good sensitivity, high detection precision, fast responsivity, and excellent mechanical stability (Figure 7c–f).

**Figure 8:**
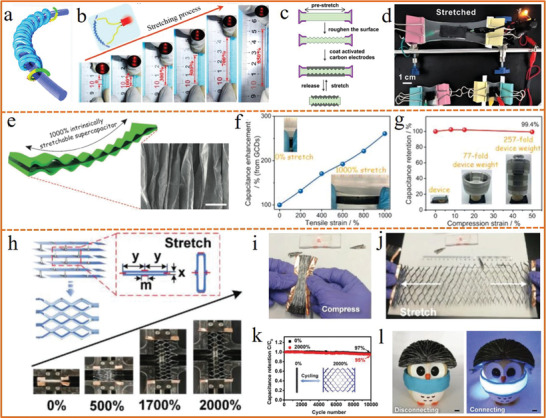
a) An illustration of an ultra‐stretchable helix structured supercapacitor. b) Photographs of the supercapacitor with tensile strain from 0% to 850% to power the light emission diode. a,b) Reproduced with permission.^[^ ^62^ ^]^ Copyright 2018, Springer Nature. c) Schematic illustration of the fabrication for stretchable supercapacitor utilizing the prestretching and release approach. d) Photographs of the LED light powered by supercapacitor devices at stretched states (*ε* = 150%). c,d) Reproduced with permission.^[^ ^184^ ^]^ Copyright 2019, American Chemical Society. e) Supercapacitor with 1000% ultra‐stretchability. f) Capacitance enhancement with strain up to 1000%. g) Capacitance retention with increase of compression strain. e–g) Reproduced with permission.^[^ ^185^ ^]^ Copyright 2017, Wiley‐VCH. h) Images of the supercapacitor under different tensile strains. i) The supercapacitor powering a red LED under compression. j) The supercapacitor powering a red LED under stretching. k) Capacitance retention of the supercapacitor during repeated tensile strain of 2000%. l) The arch bridged supercapacitor acting as a helmet worn on the head of an owl toy model to power a 3 V flexible LED strip. h–l) Reproduced with permission.^[^ ^186^ ^]^ Copyright 2018, Wiley‐VCH.

For the wire‐shaped energy storage device with high stretchability,^[^ ^180^ ^]^ the helix structural design on climbing stems was proved to be efficient. For the planar configurations, the worm‐like folds or wave structure design would make them stretchable by creating wrinkle or wavy electrode via pre‐strain or self‐twist.^[^ ^181^ ^]^ Besides, kirigami is also considered as an available route to turn 2D paper into deformable 3D structure with high stretchability.^[^ ^182^, ^183^ ^]^ For instance, two flexible CNT@graphene@MnO_2_ fibers with helical structure around a super elastic core fiber to assemble an ultra‐stretchable wire‐shaped supercapacitor with tensile strain up to 850% (**Figure** **8**a,b).^[^ ^62^ ^]^ A tough hydrogel with roughened microstructured surface and high surface area was generated by pre‐stretching, followed by coating of activated carbon electrodes on both sides, which could serve as the solid‐state polymer electrolyte and the stretchable supporting substrate simultaneously (Figure 8c).^[^ ^184^ ^]^ The tough hydrogel exhibited an improved interfacial property between electrodes and electrolyte, resulting in the stretchable supercapacitor with enhanced electrochemical performance as well as mechanical stability (Figure 8d).

In addition to stretchability, compressibility is also an essential feature for flexible devices. Our group^[^ ^185^ ^]^ fabricated an ultra‐stretchable and compressible polyelectrolyte comprised of polyacrylamide (PAM) hydrogel cross‐linked by vinyl hybrid silica nanoparticles (VSNPs). When strain was applied in the polyelectrolyte, the cross‐linkers could serve as buffers to dissipate energy, which contributed to its high stretchability and compressibility. Thus, a supercapacitor with the afore‐mentioned wavy electrode was fabricated by paving polypyrrole (PPy) electrodes on both sides of the stretched electrolyte and then releasing the strain. The fabricated supercapacitor can be stretched up to an unprecedented 1000% strain with enhanced capacitance and compressed to 50% strain with well retained capacitance (Figure 8e–g), providing a promising route for the fabrication of intrinsically stretchable and compressible electronics. A 3D stretchable supercapacitor has been developed by Lv et al.^[^ ^186^ ^]^ with the electrodes composed of PPy/black‐phosphorous oxide electrodeposited on a highly flexible CNT film. As shown in Figure 8h, it could be stretched up to 2000% strain without structure failure, which also displayed stable function with the connected LED remaining lightened during compression and ultra‐stretch (Figure 8i,j). Take the 1 cm thick rectangular‐shaped stretchable supercapacitor, for example, it delivered ultrahigh specific areal capacitance of 7.35 F cm^−2^ and a 95% capacitance retention after 10 000 cycles of 2000% stretch‐and‐release (Figure 8k). Besides, it could be developed to an arched bridge shape, serving as a helmet worn on the head of a toy model to power a 3 V flexible blue LED strip (Figure 8l).

**Figure 9:**
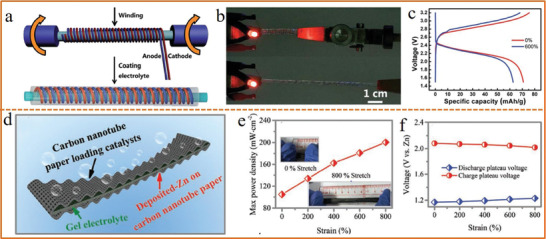
a) Schematic illustration of a stretchable fiber‐shaped battery. b) Photograph of the battery being used to power a red LED before and after 200% stretching. c) Charging/discharging curves of the battery before and after 600% stretching. a–c) Reproduced with permission.^[^ ^193^ ^]^ Copyright 2014, The Royal Society of Chemistry. d) Schematic illustration of a stretchable planar‐shaped Zn–air battery. e) Max power density curves under different tensile strains. f) Charging/discharging voltage plateau at different tensile strains. d–f) Reproduced with permission.^[^ ^194^ ^]^ Copyright 2019, Wiley‐VCH.

Despite the fact that stretchable and compressible supercapacitors have been fully achieved,^[^ ^187^, ^188^, ^189^ ^]^ whereas the investigation of corresponding batteries are relatively sluggish due to the lack of a stretchable, compressible, and conductive electrolyte as well as the current collector with satisfied mechanical and electric properties,^[^ ^190^ ^]^ currently, two primary types of stretchable batteries, the fiber‐shaped and the planar‐shaped, have been developed via special device configuration with challenges settled.^[^ ^191^, ^192^ ^]^ A super‐stretchy, fiber‐shaped lithium‐ion batteries are fabricated by winding the highly aligned cathode and anode fibers on an elastomer substrate, followed by coating with gel electrolyte layer (**Figure** **9**a).^[^ ^193^ ^]^ The fiber‐shaped battery could continually power a red LED under both initial and stretched state (Figure 9b). More importantly, it delivered a high specific capacity of 91 mAh g^−1^ and maintained 88% initial capacity after 600% stretch (Figure 9c). Nevertheless, the electrode and the gel electrolyte of fiber‐shaped stretchable batteries are still almost unstretchable, which have to solely rely on elegant structural designs that are not applicable to the planar‐type batteries. Therefore, exploration of stretchable electrolyte with favorable ionic conductivity is also urgent for the realization of intrinsically stretchable batteries. Recently, a super‐stretchable alkaline‐tolerant PANa–cellulose dual network hydrogel electrolyte was developed by Ma et al.,^[^ ^194^ ^]^ which was successfully applied in a planar‐shape zinc–air battery with wavy electrolyte structure (Figure 9d). When the battery was stretched, the power density enhanced from 108.6 to 210.5 mW cm^−2^ that benefited from the increased contact areas between active materials and the hydrogel electrolyte (Figure 9e). Moreover, the discharge plateau moved up while the charge plateau dropped with the increase of tensile elongation, further indicating the enhancement in energy efficiency (Figure 9f). The intrinsically stretchable electrolyte developed in this work made a key progress in the field of 2D stretchable batteries toward enhanced mechanical durability and reliability.

**Figure 10:**
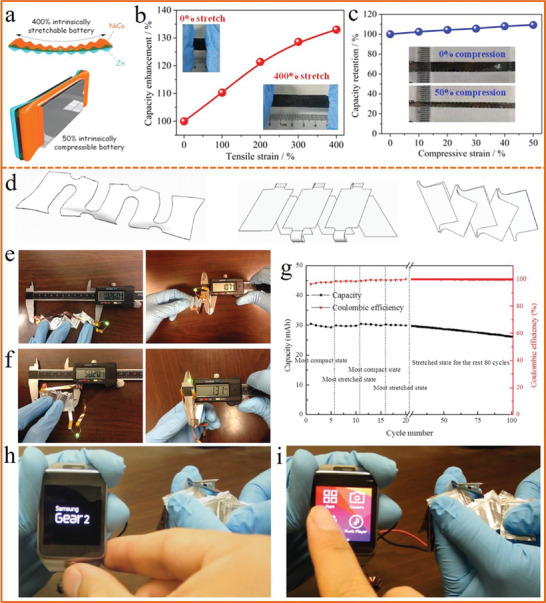
a) Schematic illustration of the NiCo//Zn battery under 400% tensile strain and 50% compressive strain. b) Capacity enhancement ratio under tensile strain. c) Capacity retention under different compressive strain. a–c) Reproduced with permission.^[^ ^196^ ^]^ Copyright 2019, Elsevier. d) Schematics of three kirigami patterns. e) Photograph of the battery under its fully stretched state. f) Photograph of the battery under its fully compact state. g) Capacity and coulombic efficiency under alternative states of compact and stretched states. h) The battery powering a smart watch at the compact state. i) The battery powering a smart watch at the stretched state. d–i) Reproduced under the terms of the CC‐BY Creative Commons Attribution 4.0 International License (https://creativecommons.org/licenses/by/4.0).^[^ ^197^ ^]^ Copyright 2015, Springer Nature.

Further, the exploration of stretchable and compressible electrolyte was also rather essential for the realization of stretchable and compressible energy storage.^[^ ^195^ ^]^ Our group fabricated a stretchable and compressible wavy‐structured NiCo//Zn battery (**Figure** **10**a) for the first time,^[^ ^196^ ^]^ which could be not only intrinsically stretched up to 400% strain (Figure 10b), but also compressed to 50% strain with capacity enhanced (Figure 10c). Moreover, the battery exhibited stable electrochemical performances even after being stretched for 500 cycles and compressed for 1500 cycles with 87% and 97% of its initial capacity retained, respectively. Additionally, kirigami is also proved to be efficient to achieve high stretchability and compressibility for flexible energy storage. Figure 10d presents three kirigami patterns with the ability to be stretched and compressed: zigzag‐cut, cut‐N‐twist, and cut‐N‐shear. Song et al.^[^ ^197^ ^]^ fabricated a cut‐N‐shear pattern LIB with stretchability higher than 150%. More significantly, the stretchable planner battery could be achieved simply by conventional components (graphite as the anode and LiCoO_2_ as the cathode) and the traditional battery manufacturing (slurry coating) and packaging procedures. It was just folded and cut with stretchability achieved based on the particular kirigami pattern. As shown in Figure 10e,f, there was no significant change on the thickness of the battery at the most stretched state and most compact state. Moreover, the battery presented stable electrochemical performance up to 100 cycles under alternative states of compaction and stretch (Figure 10g), and powered a smart watch under these states (Figure 10h,i). Such structural design concept in this work could also be expanded to other devices by offering extra physical and functional design spaces.

### 3.3 Biodegradable Flexible Energy Harvesting and Storage: To Nature

The pollution induced by electronic wastes and the toxic/harmful effect of electronic materials on our human body should be paid attention for the sustainable development of both flexible electronics and environment. To this end, biodegradable or bioresorbable energy harvesters and storage are critically responsible to alleviate the environmental and demic burden induced by e‐waste, with the ability to be dissolved, decomposed, or adsorbed and thus go to the nature after finishing their missions.^[^ ^198^, ^199^, ^200^, ^201^, ^202^ ^]^

**Figure 11:**
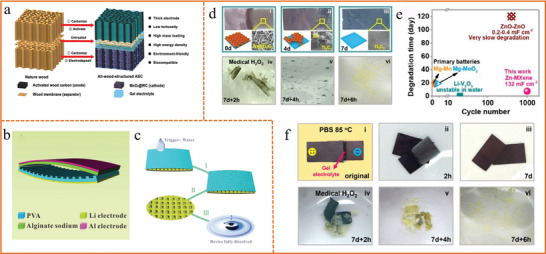
a) Schematic illustration of an all‐wood supercapacitor. Reproduced with permission.^[^ ^209^ ^]^ Copyright 2017, The Royal Society of Chemistry. b) Schematic diagram and dissolution mechanism of the recyclable and green triboelectric nanogenerator. c) Dissolution process of each component. b,c) Reproduced with permission.^[^ ^210^ ^]^ Copyright 2017, Wiley‐VCH. d) Digital, SEM, and schematic images of Zn degraded in PBS at 85 °C and digital images of the morphological evolution of Ti_3_C_2_ film triggered by medical H_2_O_2_. e) Digital images of the degradation process of Zn−MXene supercapacitor in PBS. External stimulus of medical H_2_O_2_ was added after the full degradation of Zn. f) Comparisons of degradation time and cycle number. d–f) Reproduced with permission.^[^ ^212^ ^]^ Copyright 2019, American Chemical Society.

The biodegradability of flexible energy harvesters and storage can be realized by biodegradable materials derived from the nature,^[^ ^203^, ^204^, ^205^ ^]^ soluble metals,^[^ ^206^ ^]^ and biodegradable polymers,^[^ ^207^, ^208^ ^]^ which could disappear completely over a prescribed time by a controlled external stimuli including light, temperature, pH, and humidity to initiate the dissolution and degradation of electronics components. Chen et al.^[^ ^209^ ^]^ demonstrated an all‐wood asymmetric supercapacitor (ASC) based on activated wood carbon (AWC) as anode, thin wood membrane as separator and MnO_2_/wood carbon (MnO_2_@WC) as cathode (**Figure** **11**a). The all‐wood ASC device could be biodegradable in the environment and deliver remarkably high capacitance of 3.6 F cm^−2^, mainly attributed to the carbonized and directly channeled wood with high electronic and ionic conductivities as well as the low tortuosity. Zhang et al.^[^ ^210^ ^]^ demonstrated a soluble, recyclable, and green triboelectric nanogenerator based on triboelectrification and cascade reactions, which could efficiently harvest mechanical energy from the environment such as vibration and heart beating, but also could be fully dissolved and degraded within minutes triggered by water (Figure 11b,c).

The ever‐reported degradable energy storages are relatively scarce because it is very difficult to balance the degradability and the energy storage capability since few degradable components could be stable for energy storage simultaneously.^[^ ^211^ ^]^ Recently, microstructure engineering strategy was employed by Yang et al.^[^ ^212^ ^]^ to realize the all‐component degradable and rechargeable Zn–MXene supercapacitor with short degradation time but long service lifespan. The degradation rate of Zn anode can be tailored by constructing a 3D interconnected microstructure to effectively increase the contact area between Zn and phosphate buffer saline (PBS), which shortens the degradation duration from >30 to only <8 days (Figure 11d). The integrated Zn–MXene supercapacitor, consisting of degradable Zn anode, Ti_3_C_2_ film cathode degraded by external stimulus of medical H_2_O_2_, and gel electrolyte dissolvable in PBS, demonstrated complete degradation in less than 8 days (Figure 11e). Meanwhile, this Zn–MXene capacitor consisting of Zn@Ti_3_C_2_ anode, Ti_3_C_2_ cathode, and gel electrolyte is evidenced to exhibit a good cycling performance with capacitance retention of 82.5% after 1000 cycles at 3 A g^−1^. The short degradation time (7.25 days) yet long cycle number (1000) of this Zn–MXene supercapacitor surpasses all other degradable supercapacitors or batteries (Figure 11f).

**Figure 12:**
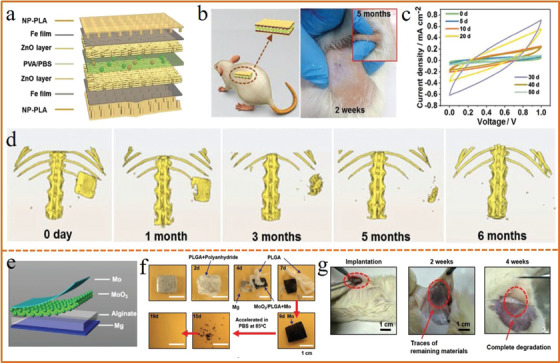
a) Structure of the as‐fabricated bioabsorbable supercapacitor. b) Implantation in the dorsal subcutaneous region of a SD rat (left) and pictures of the implanted site after different degradation time (right). c) CV curves of the implanted supercapacitor up to 50 days. d) In vivo biodegradation of the supercapacitor in a SD rat up to 6 months via micro‐CT imaging. a–d) Reproduced with permission.^[^ ^66^ ^]^ Copyright 2019, Wiley‐VCH. e) The schematic illustration of the biodegradable battery. f) Optical images of the battery at various dissolution stages in PBS. g) In vivo degradation of the battery in the subcutaneous area of SD rat. e–g) Reproduced with permission.^[^ ^219^ ^]^ Copyright 2018, Wiley‐VCH.

Bioabsorbable energy harvesters and storage are promising power source for biomedical implanted electronics with the advantage of eliminating the second surgery for device retrieval and the potential infection risk induced by long‐term implants.^[^ ^213^, ^214^ ^]^ It typically adopts water‐soluble transition metals as conductive electrodes or current collectors such as magnesium (Mg), zinc (Zn), tungsten (W), iron (Fe), molybdenum (Mo), etc., and biodegradable polymers as substrate materials such as silk,^[^ ^203^ ^]^ poly(glycerol sebacate) (PGS),^[^ ^215^ ^]^ polycaprolactone (PCL),^[^ ^216^ ^]^ poly(lactic acid) (PLA),^[^ ^217^ ^]^ polylactic‐*co*‐glycolic acid (PLGA),^[^ ^201^ ^]^ poly(1,8‐octanediol‐*co*‐citrate) (POC),^[^ ^218^ ^]^ etc. To explore in practical applications as temporary source supply in vivo, Li et al.^[^ ^66^ ^]^ developed a fully bioabsorbable supercapacitor for life‐time implantation with a symmetrical layer‐by‐layer structure, including PLA nanopillar arrays, self‐assembled zinc oxide nanoporous layer, and PVA/PBS hydrogel (**Figure** **12**a). The as‐fabricated device was implanted in the dorsal subcutaneous region of a Sprague–Dawley (SD) rat with the wound healed well without obvious inflammation after 2 weeks (Figure 12b), showing good capacitive performance in vivo up to 50 days (Figure 12c). After 5 months, the implanted supercapacitor disappeared as observed by the real‐time shape change in the SD rat monitored by micro‐computed tomography (micro‐CT) system (Figure 12d). Huang et al.^[^ ^219^ ^]^ fabricated a high‐performance fully bioresorbable primary Mg//MoO_3_ battery which consisted of all biodegradable components including Mg, MoO_3_, Mo, sodium alginate hydrogel, PLGA, and polyanhydride encapsulation layer (Figure 12e). This battery provided a 1.6 V high and stable output voltage with prolonged lifetime up to 13 days that could satisfy the requirement of implantable electronics (Figure 12f), degraded both in vitro and in vivo with desirable biocompatibility (Figure 12g). These fully bioabsorbable energy storage provide a promising energy solution to implantable therapeutic and diagnostic electronics.

**Figure 13:**
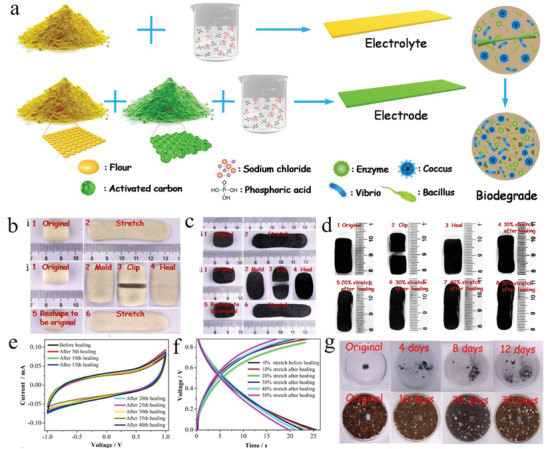
a) Fabrication of electrolyte and electrode for the flour‐based one‐stop supercapacitor. b) The stretching and self‐healing behavior of the electrolyte. c) The stretching and self‐healing behavior of the electrode. d) Photographs of the supercapacitor undergoing healing and different tensile strains after healing. e) CV curves from 0th healing to 40th healing. f) GCD curves of the supercapacitor from 0% to 50% stretch after healing. g) The biodegradation process of the supercapacitor in the simulated gastric fluid and nutritional soil. a–g) Reproduced with permission.^[^ ^220^ ^]^ Copyright 2018, Elsevier.

This section focuses on the flexible energy devices (the essential driving force for flexible electronics), and summarizes three main types of flexible energy harvesting and storage devices: self‐healing, stretchable/compressive, and biodegradable/absorbable from three perspectives of from nature, beyond nature and to nature, whereas all the works reviewed above are independent. For example, the self‐healing electronic devices could function properly after cutting but cannot be stretched because the electrode itself cannot restore the physical integrity. The stretchable/compressible device typically is not biodegradable/absorbable to the nature, etc. In such case, the realization of flexible energy device with intrinsic self‐healability, stretchability after self‐healing, and biodegradability after full usage, is rather significant for advanced modern flexible electronics. Recently, our group^[^ ^220^ ^]^ innovatively employed the self‐healable flour obtained from nature as the main component of both electrolyte and electrodes to fabricate the one‐stop supercapacitor for the first time (**Figure** **13**a), which displays intrinsic self‐healability and stretchability after healing as well as biodegradability after full utilization. Benefiting from the synchronous stretchability of the electrolyte and electrodes (Figure 13b,c), the supercapacitor displayed a rather high healing efficiency around 100% during all 40 cutting/healing cycles with high coincidence in the CV curves before and after healings (Figure 13d,e). More importantly, the self‐healable supercapacitors could even be intrinsically stretched to 50% after healing (Figure 13d,f). Moreover, our supercapacitor was eco‐friendly and biodegradable since all the raw materials used including the fully biodegradable flour are non‐toxic and eco‐friendly (Figure 13g). This one‐stop supercapacitor represents the totally green electronics that derived from nature, beyond nature and to nature, which opens new opportunities for various next‐generation flexible and implantable electronics.

## 4 Flexible Sensors

**Figure 14:**
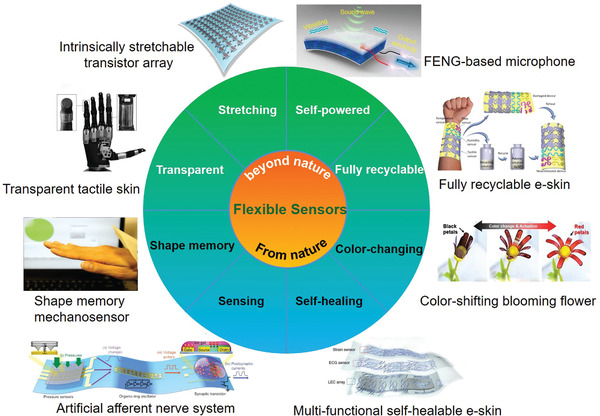
Representative flexible sensors with functionality that from nature and beyond nature. From nature: the shape memory ability was represented by the shape memory polymers based mechanosensor. Reproduced with permission.^[^ ^225^ ^]^ Copyright 2018, Wiley‐VCH. The sensing ability was represented by the artificial afferent nerve system. Reproduced with permission.^[^ ^16^ ^]^ Copyright 2018, The Authors, published by American Association for the Advancement of Science. The self‐healing ability was represented by the multi‐functional self‐healable e‐skin. Reproduced with permission.^[^ ^23^ ^]^ Copyright 2018, Springer Nature. The color‐changing ability was represented by the color‐shifting blooming flower. Reproduced with permission.^[^ ^226^ ^]^ Copyright 2018, Wiley‐VCH. Beyond nature: the fully recyclable ability was represented by the fully recyclable and self‐healable e‐skin. Reproduced with permission.^[^ ^52^ ^]^ Copyright 2018, The Authors, published by American Association for the Advancement of Science. Reprinted/adapted from ref. [52]. © The Authors, some rights reserved; exclusive licensee American Association for the Advancement of Science. Distributed under a Creative Commons Attribution NonCommercial License 4.0 (CC BY‐NC) http://creativecommons.org/licenses/by-nc/4.0/. The self‐powered ability was represented by the FENG‐based thin patch microphone. Reproduced with permission.^[^ ^227^ ^]^ Copyright 2018, Springer Nature. The stretching ability was represented by the fully patterned intrinsically stretchable transistor. Reproduced with permission.^[^ ^24^ ^]^ Copyright 2018, Springer Nature. The transparent ability was represented by the transparent tactile skin. Reproduced with permission.^[^ ^228^ ^]^ Copyright 2017, Wiley‐VCH.

Flexible sensors played a rather vital role in wearable electronics, with various intriguing functionalities that mainly derived from nature or even beyond nature (**Figure** **14**). By simulating the human skin, the multi‐functional self‐healable e‐skin was widely explored. Inspired by the sensing ability in living creatures,^[^ ^221^ ^]^ which could respond to external environmental stimuli, thus, the artificial nerve system and various environmental detection sensors were developed.^[^ ^222^, ^223^ ^]^ The color‐changing ability and shape memory phenomenon in nature have also been applied in the multi‐functional flexible sensors. Additionally, more powerful functionalities that are beyond nature were also exploited in flexible sensors, such as the fully recyclable e‐skin, the transparent tactile skin, the self‐powered microphone or e‐skin^[^ ^224^ ^]^ that cannot be found in nature, the stretchable transistor array with ultrahigh stretchability that is far beyond nature. In this section, important advances in these flexible sensors that from nature, represented by the environmental sensors, beyond nature represented by the self‐powered sensors and the biodegradable sensors to nature are comprehensively summarized with the aim to guide the design of flexible electronics toward intellectualization, multi‐functionalization, and ecological sustainability.

### 4.1 Environmental Sensors: From Nature

Nowadays, severe contamination issue caused by industrial chemicals, consumer products, and biocides threatens the security of human and other creatures. Environmental monitoring is one essential approach to detect the environment change, such as temperature,^[^ ^229^, ^230^, ^231^ ^]^ light,^[^ ^232^, ^233^, ^234^, ^235^, ^236^ ^]^ humidity,^[^ ^237^, ^238^, ^239^, ^240^, ^241^ ^]^ pH,^[^ ^242^, ^243^, ^244^ ^]^ heavy metal ion,^[^ ^245^ ^]^ volatile organics,^[^ ^246^ ^]^ gas,^[^ ^247^, ^248^, ^249^ ^]^ etc., so as to protect the green environment from deterioration. Traditional environmental sensors are bulky, rigid based on semiconductor technologies, which are far from their further application in flexible and wearable monitoring devices. In this section, the new generation of flexible environmental sensors stimulated by external nature factors, such as pH, Hg^2+^ pollution, volatile organic compound, deep‐sea environment, are described with the demand fulfillment for various application areas.^[^ ^250^ ^]^

**Figure 15:**
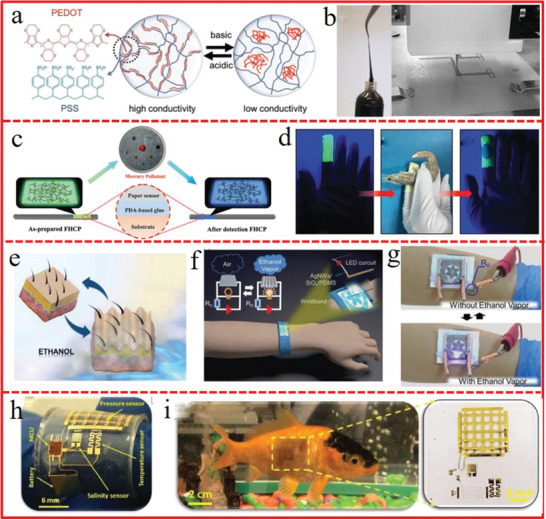
a) Schematic illustration of the impact of pH on molecular structure of PEDOT:PSS. b) Printable PEDOT:PSS/HPU inks, right is the 3D printing executed with a gel extruder on dry HPU films. a,b) Reproduced with permission.^[^ ^255^ ^]^ Copyright 2018, Wiley‐VCH. c) Schematic illustration of Hg^2+^‐sensing FHCP‐4 film sensors that are immobilized onto a wide variety of materials. d) Photos of the emission color change of the hydrogel‐coated wearable sensing gloves when exposing the forefinger tip to Hg^2+^‐polluted sea shrimp. c,d) Reproduced with permission.^[^ ^256^ ^]^ Copyright 2018, Wiley‐VCH. e) Schematic illustration of the skin surface wrinkling caused by excessive intake of ethanol. f) Schematic illustration of a smart wearable and flexible electronic device with a switchable and dynamic dual‐signal property. g) A proof‐of‐concept wearable device responds to ethanol vapor with a direct, dynamic visual and electrical feedback. e–g) Reproduced with permission.^[^ ^257^ ^]^ Copyright 2019, Wiley‐VCH. h) Performance improvement of the wearable multisensory marine skin gadget with a reliable interlocking mechanism showing all the components. i) Scaled version without rigid components adhere strongly on goldfish attached using surgical glue. h,i) Reproduced with permission.^[^ ^258^ ^]^ Copyright 2019, Wiley‐VCH.

The detection and control of pH are essential for human beings to protect the water resources from contamination.^[^ ^251^, ^252^, ^253^, ^254^ ^]^ Naficy et al.^[^ ^255^ ^]^ fabricated a highly flexible pH sensor based on poly(3,4‐ethylenedioxythiophene) (PEDOT) doped with negatively charged poly(styrenesulfonate) (PSS) and robust hydrophilic polyurethanes (HPU) (PEDOT: PSS/HPU). PEDOT: PSS was used as the sensing material while HPU as reinforcing matrix. The electrical properties of the PEDOT: PSS hydrogel varied with change in acidity or alkalinity (**Figure** **15**a,b).

The serious Hg^2+^ pollution in seafood, grain, and even drinking water make the mercury(II) detection indispensable to protect people from mercury(II)‐polluted food and water. Zhang et al.^[^ ^256^ ^]^ proposed a robust hydrophilic fluorescent hydrogel‐coated wearable chemosensor, which relied on a specific chemical reaction between Hg^2+^ and the grafted thiourea moieties with the “green‐to‐blue” emission color change (Figure 15c). On this basis, the robust fluorescent hydrogel‐coated gloves are fabricated for the first time, which effectively protect the operators from the toxic Hg^2+^‐polluted samples with the intuitive visual detection (Figure 15d). The real‐time monitoring of the volatile organic compound (VOC) explosion limit, such as ethanol, toluene, acetone, formaldehyde, and methanol, is of great significance in industrial production and environmental management. Inspired from the wrinkles formed on the skin during long‐term exposure to ethanol vapor (Figure 15e),^[^ ^257^ ^]^ a chemical molecule triggered smart wearable sensor based on AgNW/SiO*x*/PDMS multilayered film was constructed (Figure 15f), which can respond to VOCs with dual‐signal response of both dynamic visual and electrical signal feedback (transparency and resistance) (Figure 15g). This platform can be versatilely exploited for other chemical stimuli‐responsive sensors in the environmental monitoring and wearable electronics.

Deep‐sea environmental monitoring is also very important to quantify the distribution of the human impacts on the marine ecosystem, such as pollution, overexploitation, warming, and acidification. Marine skin, a flexible and stretchable multisensory platform, was developed by F. Shaikh et al.^[^ ^258^ ^]^ to monitor the temperature, pressure, and the salinity of the marine environment (Figure 15h). Due to the harsh environment and the varying conditions, an extremely rugged and robust version of the marine skin tagging platform was demonstrated with an interdigitated electrode pattern adopted to increase the reliability and sensitivity of performance under high pressure, high salinity, and reduced temperature. The flexible skin‐like multifunctional electronic tagging system can be noninvasively capable of monitoring the marine environment even at great depths with a simple attachment on the tiny goldfish (Figure 15i).

### 4.2 Self‐Powered Sensors: Beyond Nature

Flexible integrated sensors, represented by e‐skin, are regarded as the next generation wearable technology for their wide applicability in digital healthcare^[^ ^259^ ^]^ and internet of things (IoT),^[^ ^260^, ^261^ ^]^ with the need to be powered by external power source.^[^ ^262^ ^]^ The pursuit of self‐powered electronics based on energy harvesting technologies by extracting energy from the ambient environment and converting to electricity are rather intriguing for the development of compact, flexible, and wearable devices without the utilization of heavy batteries.^[^ ^263^, ^264^, ^265^, ^266^, ^267^, ^268^ ^]^ This section will review on the self‐powered sensors with various sensing functionality,^[^ ^269^ ^]^ such as light detecting,^[^ ^270^ ^]^ gas sensing,^[^ ^271^, ^272^ ^]^ motion sensing,^[^ ^273^, ^274^ ^]^ physiological signals sensing,^[^ ^275^ ^]^ etc., which were based on the energy‐harvesting technologies including photovoltaic,^[^ ^276^, ^277^ ^]^ thermoelectric,^[^ ^278^, ^279^ ^]^ piezoelectric,^[^ ^280^, ^281^ ^]^ and triboelectric effect,^[^ ^282^, ^283^, ^284^ ^]^ etc. The self‐powered ability is considered “beyond nature” as the integration of sensing and self‐powering behaviors in one single system cannot be found in living organisms of the nature.

**Figure 16:**
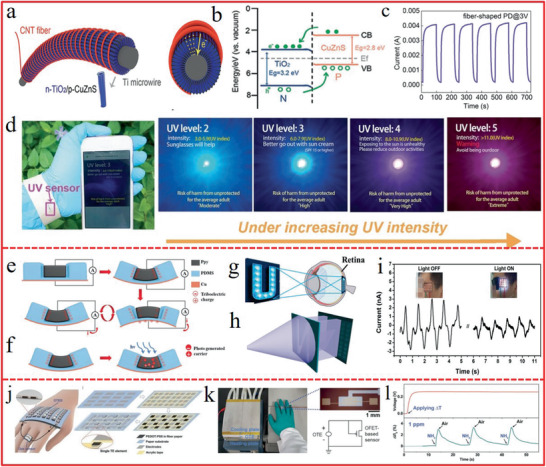
a) Schematic illustration of the device configuration of fiber‐shaped p‐CuZnS/*n*‐TiO_2_ PD. b) Band diagram of the p‐CuZnS/n‐TiO_2_ heterojunction showing the photo generated carrier transfer process under UV illumination. c) The on‐off switching tests of the fiber‐shaped PD at 3 V under 350 nm. d) A wearable real‐time UV monitoring system in real life. a–d) Reproduced with permission.^[^ ^60^ ^]^ Copyright 2018, Wiley‐VCH. e) The triboelectrification process between PDMS and PPy. f) The triboelectrification/photodetecting coupling process. g) The imaging process of retina. h) Schematic diagram of the measurement set. e–h) Reproduced with permission.^[^ ^285^ ^]^ Copyright 2018, Wiley‐VCH. i) The outputing current of the e‐skin driven by blinking eyes. j) Schematic illustration of an OSSE integrating a flexible OTE generator with a paper substrate and an OFET‐based sensor. Right is the schematic illustration of the fabrication process of an OTE generator with PEDOT: PSS legs. k) Photographs of an OTE array powered chemical sensor. The right graphs show the sensing OFET and corresponding circuit diagram for the OSSE. l) Output voltage of an OTE array driven by a temperature difference created by a heating plate and a cooling flow, and the time monitoring of the current change in response to 1 ppm ammonia of the sensing OFET powered by the OTE array. i–l) Reproduced with permission.^[^ ^286^ ^]^ Copyright 2019, Wiley‐VCH.

Noticeably, a real‐time UV light sensor is highly needed since excess UV radiation is believed to be the major cause of skin‐related cancers. A wearable UV sensor with good spectrum selectivity that is solar blind but UV sensitive should be pushed on road. Xu et al.^[^ ^60^ ^]^ developed a fiber‐shaped p‐CuZnS/n‐TiO_2_ photodetector (PD) (**Figure** **16**a), which was self‐powered due to the photovoltaic effect (Figure 16b) with high photocurrent of 4 mA (Figure 16c) that mainly benefited from the hybrid 1D nanostructure with large specific surface area. It provided better optical and electrical properties such as longer lifetime charge carrier, better charge separation, and higher carrier generation. The flexible fiber‐shaped PD could be integrated with a commercial data collector and communicator to form a real‐time UV radiation monitor (Figure 16d). The UV density can be easily read on the mobile phone, which can remind people of the UV intensity in time and protect from the risk of excessive UV exposure. As one of the important applications for the self‐powered PD, vision e‐skin with the visual‐image perception ability would make the brain–machine interaction system become possible. Dai et al.^[^ ^285^ ^]^ developed a new self‐powered vision e‐skin based on PPy/PDMS triboelectric‐photodetecting pixel‐addressable matrix (Figure 16e). Because of the triboelectrification/photodetecting coupling effect (Figure 16f), the triboelectric output signal is significantly dependent on the intensity and wavelength of the illumination, acting as the electricity power and photodetecting signal simultaneously. By mimicking the retina imaging process (Figure 16g), the e‐skin can map single‐point and multipoint illumination stimuli via the multichannel data acquisition method (Figure 16h). Moreover, the e‐skin can be driven by human motion (such as blinking eyes), and no external electricity power is needed in both photodetecting and signal transmitting processes (Figure 16i).

In addition to the abovementioned self‐powered sensors with power generated by external physical light stimuli, the construction of self‐powered sensors for chemical stimuli would enable even more fascinating applications. Recently, a flexible self‐powered hemical sensor was achieved based on an organic thermoelectric (OTE) generator to harvest energy and n‐type organic field‐effect transistor (OFET) to detect chemical stimuli (Figure 16j).^[^ ^286^ ^]^ The power generation ability is sufficient to drive a sensing OFET gas sensor with an ultralow operating voltage (Figure 16k), which exhibits a sensitive response to ammonia at the sub‐ppm level without an additional power supply (Figure 16l).

**Figure 17:**
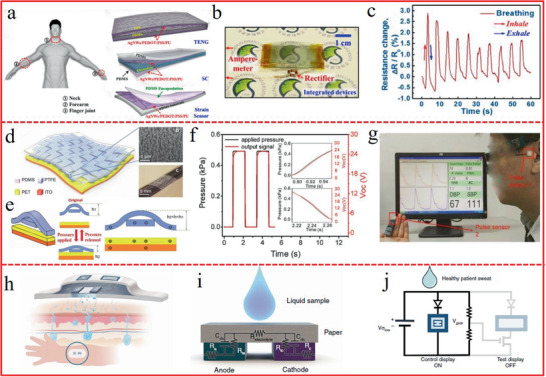
a) Schematic illustration of integrated self‐powered device with TENG, supercapacitor and strain sensor. b) Photograph of the vertically integrated self‐powered devices. c) Resistance change (Δ*R*/*R*_0_) of the nanocomposite strain sensor versus time, measured by a source measurement unit, during breathing. a–c) Reproduced with permission.^[^ ^291^ ^]^ Copyright 2015, American Chemical Society. d) Schematic illustration of the flexible weaving constructed self‐powered pressure sensor with SEM image of plasma‐etched PTFE nanowires and photograph of as‐fabricated SPS. e) Schematic diagram of the cross‐sectional view of the SPS single unit at original state and under pressure state with the electrical signal generation process illustration. f) SPS response time characterization. The response time was tested by periodically applying the pressure. g) Demonstration of the sensor system simultaneously monitoring the pulse waves from human fingertip and ear. d–g) Adapted with permission.^[^ ^292^ ^]^ Copyright 2018, Wiley‐VCH. h) Scheme of the designed patch and theoretical working scenario. i) Battery model. j) Circuit operation when healthy sweat samples are analyzed. h–j) Reproduced under the terms of the CC‐BY Creative Commons Attribution 4.0 International License (https://creativecommons.org/licenses/by/4.0).^[^ ^293^ ^]^ Copyright 2019, The Authors, published by Springer Nature.

Additionally, self‐powered patchable sensor platforms to monitor the physiological signals in human activities,^[^ ^287^ ^]^ such as pulse wave,^[^ ^288^ ^]^ sweat,^[^ ^289^ ^]^ respiration,^[^ ^290^ ^]^ etc. would bring patients comfort and convenience for real‐time health care in daily life without the adhesive electrodes or connecting wires. Lee et al.^[^ ^291^ ^]^ developed a self‐powered, all‐in‐one, patchable strain sensor that was driven by a supercapacitor charged by TENG (**Figure** **17**a,b). It could detect a wide range skin‐strain‐related human activities (breathing, coughing, drinking, swallowing, and eating) (Figure 17c), posing great prospect in personal health monitoring, soft robotics, artificial skins, etc. Meanwhile, a flexible self‐powered pressure sensor (SPS) is reported recently to precisely monitor the pulse wave and blood pressure (BP) in a noninvasive manner, which holds a multilayer structure that inspired by the common textile for clothes (Figure 17d).^[^ ^292^ ^]^ The working principle of the SPS involved the pulse‐induced membrane vibration and vibration induced electrical signal generation (Figure 17e). On one hand, the polymer nanowire enhanced‐surface roughness structure is capable of improving the surface triboelectrification and inducing higher electrical signal output. On the other hand, the woven structure greatly boosts the effective contact area and electric signals, resulting in an ultrasensitivity and an ultrafast response time of less than 5 ms (Figure 17f). As a demonstration, two wearable low power consumption sensor systems were worn against over fingertip and ear to detect human pulse waves with the results spontaneously read from the personal electronics (Figure 17g).

In addition to the abovementioned biomechanical energy, the patchable sensors stimulated by sweat with chloride ions detection also offer many ergonomic advantages with promising application in cystic fibrosis monitoring (Figure 17h). Ortega et al.^[^ ^293^ ^]^ presented a self‐powered skin patch sensor to measure sweat conductivity, which consists of a paper battery that is activated upon absorption of sweat body fluid acting as the battery electrolyte. In this sense, a battery sensor was developed with the sensor and battery merged into a single element since the generated output power and voltage is directly connected with the conductivity of the analyzed sweat (Figure 17i). The generated power enables the discerning between a healthy condition and a non‐healthy condition, with the test display activated when the battery yields a voltage that is equal to or above the cystic fibrosis‐positive threshold voltage (Figure 17j). This electrolyte content evaluation in sweat patch can also be potentially utilized for other purposes in the clinical sector, such as dehydration that is induced by physical activity.

### 4.3 Biodegradable Sensors: To Nature

**Figure 18:**
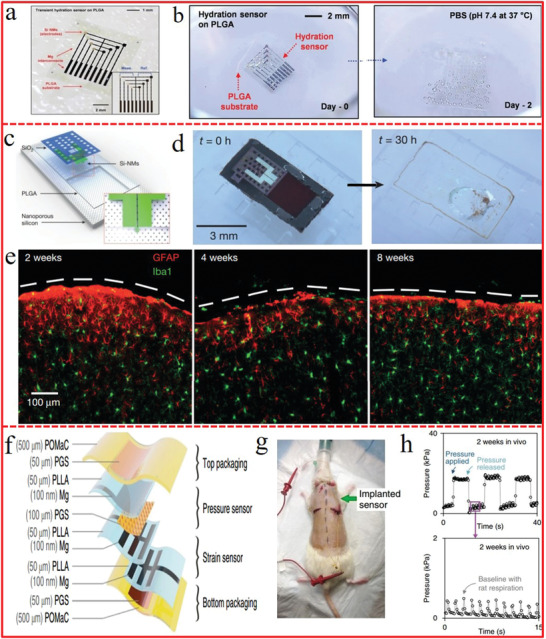
a) Image of a transient hydration sensor. b) Images of the transient hydration sensor before and after dissolution for 2 days, respectively. a,b) Reproduced with permission.^[^ ^296^ ^]^ Copyright 2014, Wiley‐VCH. c) Schematic illustration of a biodegradable pressure sensor. The inset shows the location of the Si nanowire strain gauge. d) Images of accelerated dissolution in a buffer solution (pH = 12) and a transparent PDMS enclosure at room temperature. e) Confocal fluorescence images of the cortical surface beneath the dissolved device at 2, 4, and 8 weeks, with glial fibrillary acidic protein (GFAP) to detect astrocytes (red), and ionized calcium‐binding adaptor molecule 1 (Iba1) to identify microglia/macrophages (green). The dashed line indicates the site of the implant. c–e) Reproduced with permission.^[^ ^300^ ^]^ Copyright 2016. Springer Nature. f) Schematic diagram of the fully biodegradable strain and pressure sensor. g) Implanting the sensor on the back of a Sprague–Dawley rat. h) Pressure signal detection after 2 weeks of sensor implantation. f–h) Reproduced with permission.^[^ ^301^ ^]^ Copyright 2018, Springer Nature.

For electronic products, the way to return to nature is mainly biodegradable and bioabsorbable.^[^ ^294^ ^]^ This section will focus on biodegradable/bioabsorbable sensors or their self‐powered systems in terms of material, structure, biodegradable, and sensing performance. Biodegradable sensors have been broadly researched in the aspects of biomedical and clinical surgery applications, which not only possess the fundamental sensing ability, but also can be partly or even completely biodegraded after its service lifetime.^[^ ^295^ ^]^ Being similar to the energy devices abovementioned, degradable substrate materials mainly include PLGA, PLA, PGA, PCL, and rice paper, etc. Hwang et al.^[^ ^296^ ^]^ fabricated a transient hydration sensor on a PLGA substrate to monitor healing processes at the site of a wound on the skin. **Figure** **18**a displays the image of the sensor, and an exploded‐viewed schematic illustration. The sensor was constituted of PLGA substrate, phosphorous doped Si electrodes, plasma‐enhanced SiO_2_ dielectric interlayer and Mg contacts/interconnects. Hydration levels measured by the sensor were almost identical to those observed previously in non‐transient epidermal sensors. Furthermore, the sensor was fully biodegradable with the exposed magnesium (2nd Mg) and the underlying Mg (1st Mg) dissolved first (Figure 18b), the Si and SiO_2_ dissolved completely in days or weeks, and the PLGA dissolved over months after the device was immersed in PBS (1 m, pH = 7.4) at the physiological temperature (37 °C).

Additionally, bioabsorbable sensors could be used as implantable monitoring electronics to detect physiological signals sensitively and precisely.^[^ ^297^ ^]^ Moreover, the implanted device would degrade in body, which could also eliminate the need for a second surgery to remove the device.^[^ ^298^, ^299^ ^]^ Figure 18c displays a bioresorbable pressure sensor with a magnified illustration of the active region.^[^ ^300^ ^]^ The structure involves a 30 µm PLGA membrane on top of a supporting substrate of nanoporous silicon with a square of trench etched onto the surface. The pressure responses of this sensor agreed quantitatively with those measured by clinical‐standard, non‐bioresorbable sensors. Figure 18d displays the progress of accelerated dissolution of the sensor in an aqueous buffer solution (pH = 12). The Si nanowire and SiO_2_ components were dissolved within 15 h and nanoporous Si disappeared within 30 h. When the bioresorbable sensor was implanted in live animals, immunohistochemical studies of brain tissues after implantation (2, 4, and 8 weeks) demonstrated that both the sensor and degraded by‐products were biocompatible, and no overt immune reaction was observed at the implantation site (Figure 18e).

For implantable electronics with clinical practice, a stretchable, biocompatible, and biodegradable sensor is even more favorable. Bao's group^[^ ^301^ ^]^ designed a real‐time monitoring sensor for mechanical forces on tendons with a flexible strain sensor stacked with a pressure sensor to discriminate strain and pressure stimuli with excellent sensitivity. Figure 18f shows the real picture of the assembled sensor. All the materials used in the sensor were proved to be biocompatible and biodegradable. An experiment of implanting the biodegradable strain and pressure sensor on the back of a Sprague–Dawley rat was conducted to verify the function and biocompatibility of the sensor inside the body (Figure 18g). As shown in Figure 18h, the respective similar curves of pressure and strain signals after implantation for 2 weeks indicated a stable working performance inside the body. Expectedly, no long‐term adverse inflammatory reaction was observed on the rat after 8 weeks of implantation, indicating the good biocompatibility of the sensor. The excellent biocompatibility and biodegradability of the implantable pressure and strain sensor further illustrate the potential applicability in real‐time monitoring of tendon healing.

**Figure 19:**
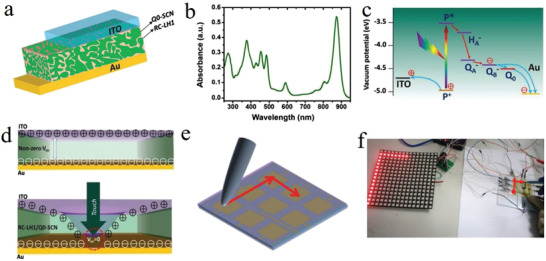
a) Device architecture (ITO‐PET/RC‐LH1/Q0‐SCN/Au‐PET). b) Absorption spectrum of the RC‐LH1 pigment‐protein in solution. c) Energy diagram showing how photo excitation of the RC‐LH1complex (rainbow arrow) elicits an intraprotein charge separation (red arrows) and direct or Q0‐mediated charge transport to the electrodes (cyanarrows). d) The blend supports a VOC between the PET‐ITO and PET‐Au electrodes. e) Schematic of a touch stimulus moving continuously along the multipixel sensor. f) Touch tracking during tracing an L‐shape on the nine‐pixel sensor. a–f) Reproduced with permission.^[^ ^305^ ^]^ Copyright 2018, Wiley‐VCH.

The self‐powered flexible sensors summarized above with various but individual sensing functionality have revolutionized the human life with driving force derived from the mechanical energy^[^ ^302^, ^303^ ^]^ or thermal energy^[^ ^304^ ^]^ in ambient atmosphere. The design of self‐powered sensor with inspiration from nature, the multiple perception ability beyond nature and the biodegradability to nature would be powerful demonstration for the beneficial effect of nature on the modern technology progress. Inspired from the natural plants, such as the “touch‐me‐not” (Mimosa pudica) and “Venus flytrap,” a self‐powered sensory electronic skin incorporated with phototransduction and photosensory functions was explored by Ravi et al.^[^ ^305^ ^]^ which far extend the sensory abilities of human skin (**Figure** **19**a). The photosynthetic protein was the Rhodobacter sphaeroides RC‐LH1 reaction center/light harvesting complex with multiple absorbance bands between 200 and 950 nm (Figure 19b,c). Touch sensing was based on the pressure which brought the electrodes into contact at the point of touch, resulting in a localized low‐resistance path for electron flow in the device that reduced the VOC to zero (Figure 19d). The flexible, multipixel, bioelectronic sensors were capable of touch registration and tracking the position of the touch (Figure 19e,f), which are both crucial for integration of an electronic skin into soft robotics. The polysensory abilities of e‐skin with phototransduction, photosensory functions, and additional nanopower generation make it particularly promising in smart wearable sensors. Herein, the adopted photovoltaic pigment‐protein as raw material was natural and biodegradable, while more biodegradable tests for this flexible bioelectronic device would be favorable to fulfill the green electronics that are totally from nature, beyond nature, and to nature.

## 5 Future Perspectives and Concluding Remarks

Significant achievements in functional materials primarily originated from nature, promoted the flourishing development of flexible electronics with functionality beyond nature and device to nature. Herein, frontier advancements in functional materials, flexible energy devices, and versatile sensors are summarized with emphasis on the vital role of nature. It is encouraging to see that impressive functionalities including self‐healing, stretching/compressing, self‐powering, environmental sensing, etc., have been achieved in flexible electronics that mainly derived from nature inspiration. However, some critical issues still existed in the development of flexible electronics, such as the design and fabrication of highly integrated flexible electronics system, the exploitation of multifunctional flexible fuel cells and solar cells, the balance between intrinsic performance and extended property based on functional materials derived from nature, the complete realization of green electronics that from nature, beyond nature, and to nature, etc. Herein, the critical issues and the potential directions for the further development of future flexible electronics are proposed in the following aspects.

Beyond the energy harvesters, energy storage, and sensors, electronic circuits are also the fundamental building block in a completed flexible electronic system, where wide bandgap semiconductors are the key components.^[^ ^306^ ^]^ Compared with traditional silicon complementary metal–oxide–semiconductor transistors, the flexible integrated circuit based on thin‐film transistor (TFTs) technologies have been the research focus for wearable electronics for their ability to be manufactured onto a variety of flexible substrates, thereby creating ultrathin, lightweight, and ultra‐flexible electronics and posing potential applications in the Internet of Things and lightweight wearable electronics.^[^ ^307^ ^]^ For the realization of ultimate “green” electronics, the exploration of biocompatible and biodegradable organic field‐effect transistors based on natural dielectrics, or nature‐inspired semiconductors were regarded as a promising route, whereas it is still in its infancy.^[^ ^308^ ^]^ Extensive biodegradable and biocompatibility studies on the nature‐inspired organic circuits should be further performed to prove the feasibility and practicability.

Over the last decade, multifunctional flexible electronics components including energy harvesters and storage, sensors, electric circuits with improved performance have been continuously developed, all of which further promote the development of integrated electronics systems, represented by the highly intelligent electronic skin (e‐skin) with broad applications in artificial intelligence, health monitoring, and human–machine interactions.^[^ ^245^ ^]^ Similar to the human skin's sensory ability, e‐skins should sense and respond to variations in the external environment including the multi‐modal force sensing, temperature, humidity detection in addition to the self‐healing and stretchable abilities.^[^ ^309^ ^]^ Future opportunities lie in the design and fabrication of highly integrated e‐skin system with high resolution, high sensitivity, and rapid response that even beyond human sensory ability. For instance, self‐powered e‐skins would be an ultimate approach to settle the power consumption issues of pressure/tactile sensors in integrated and practical e‐skin systems.^[^ ^310^ ^]^ The combination of wireless technology with e‐skins provide the foundation for integrated wearable devices in human–machine interactions for convenient sensing and energy/data transfer to monitor human body health.

Presently, rechargeable batteries are the primary energy storage device in the electronics market, whereas the exploitation of multifunctional flexible batteries such as self‐healable, stretchable/compressible, is much fewer than the supercapacitors, which might be due to the relatively complex component and critical principle for the battery. That means, at least one constitutional unit, either the electrode, electrolyte, or current collector, should be self‐healable or stretchable/compressible in addition to their fundamental functionality of energy storage, high ionic, and electronic conductivity. The advent of novel hydrogel electrolyte promotes the further realization of intrinsically self‐healable, stretchable/compressible batteries, while the investigation of multifunctional flexible electrodes should also be pushed on the road. The multiscale hierarchical structure design with the combination of charge transport and mechanical properties would be a versatile option.^[^ ^311^ ^]^

In addition, the nature‐related flexible fuel cells or solar cells with intriguing functionality are even less. In fact, there are many electrocatalysts whose morphology or property design are derived from the amazing nature. For example, inspired by the phenomenon that aquatic arachnids with hydrophobic hairs could catch air and breathe underwater,^[^ ^312^ ^]^ a similar multi‐scale hydrophobic surface of dendritic Cu catalyst was introduced to capture carbon dioxide gas with the reduction Faradaic efficiency significantly increased to 86%. Despite that, the flexible fuel cells and solar cells based on nature inspiration are still in the early stages of development. Recently, our group^[^ ^313^ ^]^ produced a flexible ethanol fuel cell for the first time with drop‐and‐play function while the investigation of flexible fuel cells has not yet reached the level of nature. In general, the progress of multifunctional flexible fuel cells and solar cells should speed up and comprehensive consideration should be provided at the beginning to achieve the flexible green energy devices with optimized structure and efficiency, including how to make use of materials from the nature, how to achieve the function beyond nature, and how to degrade the electronics to nature.

In order to realize the development of green and sustainable electronics, special materials or structures from nature should be adopted to realize the desired properties for flexible electronics that beyond nature and to nature, such as self‐healing, high stretchability, degradability, etc., which might be not the best option to realize their basic properties of energy harvesting/storage and sensing, etc. Therefore, it is not easy for these flexible electronic devices to balance the basic performance with extended functionality in all aspects, and it is more difficult to achieve simultaneous optimization. Therefore, further attempts should be engaged in developing more appropriate natural sources with optimized property to achieve the desired functions for green flexible electronics. The comprehension of the structure‐property‐function relationships in the natural materials and engineering strategy will be indeed beneficial to facilitate the nature‐inspired technology development.

A perfect modern green electronic would be realized if a flexible electronic device or system can completely follow the line of from nature, beyond nature, and to nature, which would also be the ultimate goal for the sustainable development of advanced technology with environment. Unfortunately, most of the research work is just one step in the process. At present, only the reported work on one‐stop supercapacitor by Hu et al.^[^ ^220^ ^]^ realizes this completely green nature process, and the other work of flexible energy or sensor has not appeared. Thus, there still exists a lot of work on the green electronics design need to put forward in the line with nature, which will play a favorable impact in the future.

Undoubtedly, nature is a rich source of inspiration and provides us wise route to fabricate more advanced green flexible electronics with more new functionalities that are even beyond nature, while the investigation on nature‐inspired flexible electronics is still at the initial stage when the overall performance should be enhanced and many other energy or sensors need to be explored. We believe that the continuous consideration and solution of these challenges will further realize the ultimate goal of sustainable development in flexible electronics technology and human society.

## Conflict of Interest

The authors declare no conflict of interest.
